# Institutionalization of Projects Into Districts in Low- and Middle-Income Countries Needs Stewardship, Autonomy, and Resources

**DOI:** 10.9745/GHSP-D-20-00170

**Published:** 2020-06-30

**Authors:** Peter Waiswa

**Affiliations:** a Maternal, Newborn, and Child Health Centre of Excellence, Makerere University School of Public Health, Kampala, Uganda.; b Department of Global Public Health, Karolinska Institutet, Solna, Sweden.

## Abstract

Important attributes for project institutionalization include strong stewardship and champions, affordability, demand for the intervention and perceived benefit, minimal complexity, and optimal intervention design and period of support.

## Key Messages

- There are too many projects in developing countries; ill health continues because most projects fail to be institutionalized.
- Although a district-led integrated community case management project in Uganda had donor support, sufficient implementation led by a nongovernmental organization, and adequate human resources, it had no national or district budget for commodities and lacked supervision and monitoring. All these are characteristics of a failed design for institutionalization.
- Institutionalization requires optimal design with stewardship, autonomy, capacity, affordability, minimal complexity, and a system for accountability.

See related article by Nanyonjo et al.

Driven by high morbidity and mortality, weak health systems, weak governance, and poverty, many countries in sub-Saharan Africa have a multitude of projects led by government, nongovernmental organizations, and researchers trying to fill gaps. Unfortunately, although the rhetoric is usually to “institutionalize” the project, many of these projects often fail in what others call “pilotitis”^1^ —a situation in which projects are first piloted but not sustained or scaled up. This is a practice that many governments claim that they are tired of, but business as usual still continues.

In this way, Uganda is no exception. As a result, despite proliferation of “high-impact” projects, child mortality remains high in Uganda, and the country is unlikely to achieve the Sustainable Development Goals related to child health. A recent United Nations report estimated that, despite marked progress, 74,000 children die every year in Uganda—the majority from preventable diseases.^2^ One of the main strategies that has the potential to make significant improvements to child health in sub-Saharan Africa is a strong district-led integrated community case management (iCCM) implementation.^3^

## INSTITUTIONALIZATION OF A DISTRICT-LED PROJECT IN UGANDA

The article by Nanyonjo et al.^4^ in this issue of GHSP reports the findings of an evaluation of their efforts to institutionalize iCCM in 9 districts of Western Uganda. Designed by the United Nations Children’s Fund and the World Health Organization, iCCM is a strategy that relies on community health workers (CHWs) using simple algorithms to offer health promotion, disease prevention, and curative services for uncomplicated diarrhea, malaria, and pneumonia. Since 2010, iCCM has been policy in Uganda, and it evolved from the Home-Based Management of Fever program and policy. Working in close collaboration with the Ministry of Health (MOH) in Western Uganda, the Malaria Consortium used a health systems strengthening approach to introduce and try to institutionalize iCCM in the districts from 2010 to 2015. They took institutionalization to mean that a health intervention becomes a routinely practiced and integral part of the conventional health system—I would like to add “without much more effort from external actors.” Their creative evaluation used qualitative research methods in 2010 to assess for “district preparedness” for institutionalization and later in 2015 to assess the outcomes of their efforts. A major limitation of their approach to evaluation is that it (1) does not include any quantitative measures of “readiness” or “institutionalization,” so it is difficult to measure the change; and (2) does not describe in detail any institutionalization activities or findings between 2010 and 2015.

Based on my decade-long work in districts and our experiences with project implementation at the Makerere University Maternal Newborn and Child Health Centre of Excellence (MNCH Centre)^5^ in Uganda, the results of their evaluation were not surprising: failed institutionalization. After 5 years of support, the districts were not able to maintain supply of medicines, supervision and motivation of CHWs, or maintenance of the reporting system. The authors attributed the poor institutionalization to lack of stewardship on how to transition from externally supported implementation to district-led programming, conflicting guidelines on community distribution of medicines, poor community-level accountability systems, and limited decision-making autonomy at the district level.

What really drives institutionalization? Similar to the Malaria Consortium, our MNCH Centre implemented the District Empowerment for Scale-up (CODES), an iCCM project in 16 high-burden districts in Uganda. CODES was designed to diagnose and resolve health system bottlenecks, primarily the challenges related to the district’s management of local health services. We found that although CODES improved uptake of child health interventions, the CODES approach was difficult to sustain (forthcoming publication). However, similar to the findings that Nanyonjo et al. report, we found that the main barriers included limited fiscal space constraining district managers’ ability to implement solutions identified through CODES.^6^^–^^9^ In another project, the Maternal and Newborn Scale-Up project, in Eastern Uganda, we aimed to improve newborn care in 6 hospitals serving 4 million people over a 5-year period. All 6 neonatal care units we started are still functional 2 years after implementation ended.^10^ We consider this to be a true institutionalization. Factors that facilitated institutionalization included: (1) strong leadership involvement and engagement; (2) the emergence of champions, (3) alignment to policy and structures, (4) use of mainly local resources, such as hospital space, no additional health workers, commodity availability from the hospital’s own resources, and routine data systems; (5) health workers’ and community’s demand and perceived benefit; and (6) implementation over a fairly long time (5 years) with full local staff and leaders’ involvement.

Unfortunately, this was not the case with the Malaria Consortium iCCM project. They report that they did not find the MOH engagement adequate. Further, the CHWs (called village health teams in Uganda) are an informal voluntary structure and are not salaried and depend on cash and other incentives. Finally, they report that there was no government/district budget for medicines or supervision, and there was a concern of giving medicines to community members with fears of wastage, affordability, and development of antimicrobial resistance. In addition, the iCCM design has additional flaws: scale-up of iCCM requires thousands of CHWs, who receive and manage medicines, treat people, and report regularly. In essence, each of these iCCM CHWs is a “health center.” Indeed, even the MOH considers them so, and they are called “health center level 1” as Nanyonjo et al. also report. These CHWs who have medicines are in addition to the thousands of other health centers that are located, on average, within 5 km of households. It is clear that such a complex program, although desirable, is not affordable or indeed sustainable, especially so by districts.

## ATTRIBUTES FOR SUCCESSFUL INSTITUTIONALIZATION

From the above, in addition to what we saw in the Maternal and Newborn Scale-Up project, here, we learn about other important attributes for institutionalization, namely: affordability, minimal complexity, and optimal intervention design. The World Health Organization and others recommend that if projects are to be institutionalized or sustained, they need to be “designed with the end in mind.” Among several presumably successful projects that failed institutionalization in Uganda is the Saving Mothers, Giving Life project. Although the project reportedly reduced maternal mortality rate by 40%, it was never sustained because it had minimal design involvement by the MOH, was costly, and was too complex for district health systems to absorb after the departure of external financing and nongovernmental organization management.^11^ Examples of successful projects that have been institutionalized in Uganda include immunization and the supply of critical antimalarial drugs and family planning commodities in health facilities.

To conclude, for Uganda’s iCCM project to be institutionalized, it must be redesigned. Such a design must include strong leadership at the center and in the districts, more use and alignment to existing structures and resources (nearby health centers with their staff) except in hard-to-reach areas, additional resources at the MOH and in districts, more district-level decision space, and marked accountability and involvement of community and district-level actors. In real terms, it means nongovernmental organizations should secede their power and resources to the districts right from the beginning and there should be a mechanism for ensuring capacity and accountability from the districts and the central government.
